# Distinct Impact of Processing on Cross-Order Cry1I Insecticidal Activity

**DOI:** 10.3390/toxins17020067

**Published:** 2025-02-03

**Authors:** Dafne Toledo, Yolanda Bel, Stefanie Menezes de Moura, Juan Luis Jurat-Fuentes, Maria Fatima Grossi de Sa, Aida Robles-Fort, Baltasar Escriche

**Affiliations:** 1 Institute of Biotechnology and Biomedicine, University of Valencia, 46100 Valencia, Spain; dafne.toledo@uv.es; 2 Department of Genetics, University of Valencia, 46100 Valencia, Spain; aida.robles@uv.es; 3 Department of Entomology and Plant Pathology, University of Tennessee, Knoxville, TN 37996, USA; smenezes@utk.edu (S.M.d.M.); jurat@utk.edu (J.L.J.-F.); 4 Embrapa Genetic Resources and Biotechnology, Brasília 70770-917, DF, Brazil; fatima.grossi@embrapa.br

**Keywords:** *Bacillus thuringiensis*, Cry1Ia processing, Mode of action, Colorado potato beetle (CPB), *Leptinotarsa decemlineata*, European corn borer (ECB), *Ostrinia nubilalis*

## Abstract

The insecticidal Cry proteins from *Bacillus thuringiensis* are used in biopesticides or transgenic crops for pest control. The Cry1I protein family has unique characteristics of being produced during the vegetative rather than sporulation phase, its protoxins forming dimers in solution, and exhibiting dual toxicity against lepidopteran and coleopteran pests. The Cry1Ia protoxin undergoes sequential proteolysis from the N- and C-terminal ends, producing intermediate forms with insecticidal activity, while in some cases, the fully processed toxin is inactive. We investigated the oligomerization and toxicity of Cry1Ia intermediate forms generated through trypsinization (T-Int) and larval gut fluid (GF-Int) treatments, as well as the fully trypsinized protein (toxin). Heterologously expressed intermediate forms assembled into oligomers and showed similar toxicity to Cry1Ia protoxin against *Ostrinia nubilalis* (European corn borer) larvae, while the toxin form was ~30 times less toxic. In contrast, bioassays with *Leptinotarsa decemlineata* (Colorado potato beetle) larvae did not show significant differences in toxicity among Cry1Ia protoxin, T-Int, GF-Int, and fully processed toxin. These results suggest that the Cry1I mode of action differs by insect order, with N-terminal cleavage affecting toxicity against lepidopteran but not coleopteran larvae. This knowledge is essential for designing pest control strategies using Cry1I insecticidal proteins.

## 1. Introduction

*Bacillus thuringiensis* (Bt) is a ubiquitous Gram-positive spore-forming bacterium producing proteinaceous parasporal bodies containing δ-endotoxins, which are active against several arthropod orders [1]. Among the δ-endotoxins, the 3-Domain (3-D) family of Cry proteins has been one of the most extensively studied and used commercially for pest control. There are two main types of 3-D Cry proteins based on their protoxin size: a group of larger protoxins (120–140 kDa in molecular weight) and those with smaller protoxins (70–80 kDa) yet processing by host midgut proteases yields an active toxin core (55–70 kDa) in both cases [2]. These 3-D Cry active toxin cores share a structure composed of three domains: Domain I is an α-helix bundle, Domain II folds in a β-prism, and Domain III displays a β-sandwich topology (reviewed in Xu et al. [3]). In addition to sharing structure, these domains contain five blocks of amino acids conserved among 3-D Cry proteins, supporting their relevance in the toxin mode of action. Activated toxins bind through Domains II and III to receptors on the surface of host midgut cells, which eventually results in toxin oligomerization, insertion on the enterocyte membrane, and formation of toxin pores that induce osmotic cell death [4]. Enterocyte death collapses the midgut epithelial barrier and favors gut bacteria invading the hemocoel to cause septicemia and death of the host [5].

Although the mechanism of 3-D Cry toxin insertion in the enterocyte membrane is not fully elucidated, the four currently considered models identify a critical role for helices in Domain I for this process (reviewed in Pacheco et al. [6]). The “umbrella” model proposed a hairpin formed by helices α4–α5 as responsible for membrane penetration while the remaining Domain I alpha helices were embedded in the membrane and the rest of the toxin remained above the cell membrane. The “buried dragon” model proposed that the regions from helices α2 to α7 in Domain I and the entirety of Domain III insert into the membrane, while Domain II remains exposed to the solvent. In contrast, the “penknife” model proposed conformational changes in α1–α3 that enable membrane insertion, while the rest of the toxin was inserted in the cell membrane and Domains II and III lined the pore. Finally, the “folding cane” model proposes α1–α3 helices as key for oligomerization as a trimer or a tetramer, followed by insertion in the membrane through an extended helix formed by α1 to α4 and with the solvent location of Domains II and III matching the umbrella model.

Members of the Cry1I family of 3-D Cry proteins present protoxins of ~80 kDa displaying dual toxicity against insects in the taxonomic orders Lepidoptera and Coleoptera [7,8,9]. Moreover, Cry1I proteins do not share midgut receptors with Cry1Ab or Cry1F toxins [10] and thus may be used for pyramiding efforts in transgenic plants to increase activity range and delay resistance [11,12,13]. However, the mode of action of Cry1I proteins is not completely known and has some peculiarities compared to other Cry1 proteins.

The proteolytic processing of the C-terminus in Cry1I protoxins produces a ~70 kDa pro-protein intermediate that after further proteolysis results in the fully activated ~50 kDa toxin, which at the N-terminus commences at R155 [14]. This residue is in the middle of the α-5 helix of Domain I, suggesting that helices from α-1 to half of α-5 in Domain I, which are proposed as essential for oligomerization and membrane insertion in 3-D Cry toxins [6], are missing in fully processed Cry1I toxins. This observation helps explain why the typical oligomeric structure found in other Cry1 proteins in the presence of midgut proteins was not detected when using Cry1Ia toxins [14]. The lack of oligomer formation explains the dramatically reduced activity of the ~50 kDa Cry1I toxin compared to the Cry1I protoxin form in the lepidopterans *Ostrinia nubilalis* [14,15] and *Plutella xylostella* [16].

The present study aimed to test the relevance of processing Cry1Ia protoxins at the N-terminus for insecticidal activity on two relevant pests belonging to different taxonomical orders (Lepidoptera and Coleoptera). The proteolytic dynamics were studied using purified Cry1Ia protoxin and intermediate forms after processing by trypsin or *O. nubilalis* gut fluids. The state of the Cry1Ia protoxin and partially processed toxins in solution was monitored to determine how the progressive proteolysis affected protein structure and mode of action. The toxicity of the Cry1Ia protoxin, intermediate, and fully activated protein forms was tested against larvae of *O. nubilalis* (Lepidoptera) and *Leptinotarsa decemlineata* (Coleoptera) previously reported as susceptible to Cry1I [9,14]. The results obtained in this study advance our understanding of the Cry1Ia mode of action and guide engineering efforts for the optimal use of Cry1I proteins in pest control.

## 2. Results

### 2.1. Expression and Purification of the Cry1Ia Intermediate Proteins

**Figure 1:**
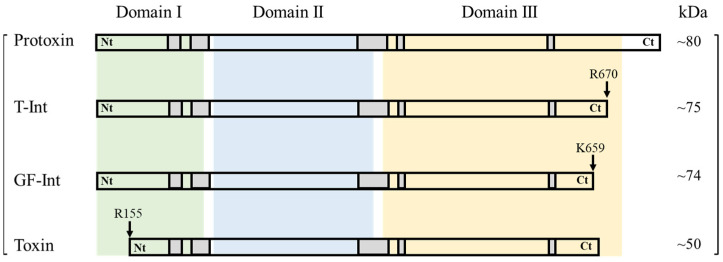
Schematic representation of the Cry1Ia38 protoxin, trypsin intermediate (T-Int), gut fluid intermediate (GF-Int), and the fully processed (Toxin) domain features and cleavage sites. Green, blue, and yellow segments represent domains I, II, and III, respectively. The grey squares represent the five conserved amino acid blocks. Cleavage sites are indicated by arrows. The Ct end in the Toxin form depends on the processing agent (trypsin or gut fluid). Modified from Khorramnejad et al. [14].

**Figure 2:**
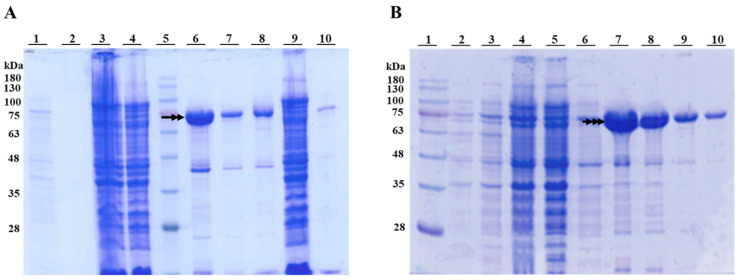
Production in *E. coli* cultures and purification of the Cry1Ia T-Int (**A**) and GF-Int (**B**) intermediary protein forms. (**A**) Lane 1: bacterial culture; Lane 2: supernatant from first culture centrifugation; Lane 3: sample after sonication; Lane 4: final supernatant; Lane 5: Blue Star molecular weight marker (Nippon Genetics Europe GmbH, Düren, Germany). Lanes 6–10 are samples from affinity chromatography purification, Lanes 6–8: eluted fractions 2, 3, and 4, respectively; Lane 9: flow-through; Lane 10: wash buffer flow-through. (**B**) Lane 1: Blue Star marker; Lanes 2 and 3: two independent bacterial cultures; Lane 4: pooled supernatant from centrifugation of both cultures; Lane 5: column flow-through; Lane 6: column wash; Lanes 7–10: eluted fractions 3, 4, 5, and 6, respectively. The numbers on the left indicate the molecular weight (in kDa) of the molecular weight marker bands. Double and triple arrows indicate the T-Int and GF-Int protein bands, respectively.

The features of the different intermediate and fully processed (“toxin”) forms compared to the Cry1Ia protoxin are shown in Figure 1. The expression of the intermediate forms from processing the Cry1I protoxin with trypsin (T-Int) and gut fluids from *O. nubilalis* larvae (GF-Int) was assessed by SDS-PAGE (Figure 2). The “toxin” form was obtained through protoxin trypsinization; however, due to the instability of the intermediate forms during trypsin processing [14], they were obtained from clones expressing sequences of Cry1Ia that had been site-directed mutagenized to produce the intermediate forms.

Total protein staining of SDS-PAGE gels detected bands of ~70 kDa for both T-Int and GF-Int purified from *E. coli* cultures, the expected molecular weight for both protein forms. Although some smaller proteins co-purified in T-Int and GF-Int samples, they represented a relatively negligible fraction of the total protein content in each sample (Figure 2).

### 2.2. Oligomeric State in Solution

**Figure 3:**
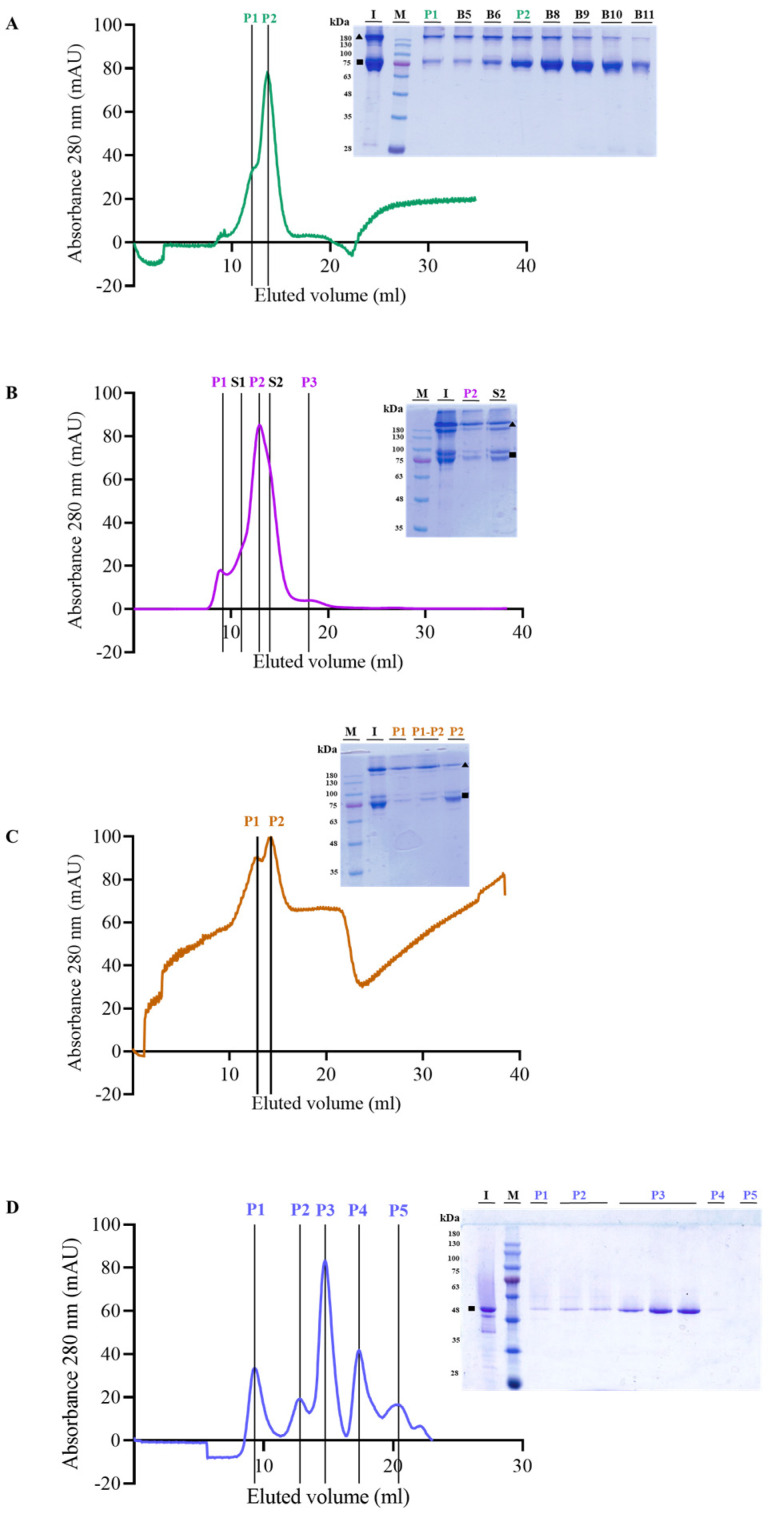
Size exclusion chromatography analysis of Cry1Ia protein forms. (**A**) Protoxin. Lanes in the gel image are I: protein before SEC analysis; M: Blue Star marker (Nippon Genetics Europe GmbH, Düren, Germany); P1: chromatogram peak 1; B5 and B6: fractions between peaks 1 and 2; P2: peak 2; B8 and B9: fractions after peak 2. (**B**) T-Int sample. Lanes in the gel image are M: Blue Star marker; I: protein before SEC analysis; P2 and S2: chromatogram peak 2 and shoulder 2, respectively. (**C**) GF-Int samples. Lanes are M: Blue Star Marker; I: protein before SEC analysis; P1: peak 1; P1-P2: fractions in between peaks 1 and 2; P2: peak 2. (**D**) “Toxin” sample. Lanes in the gel image are I: protein before SEC analysis; M: Blue Star marker; P1: peak 1; P2: the two fractions of peak 2; P3: three fractions of peak 3; P4: peak 4; P5: peak 5. Numbers on the left of the gel images indicate the size of the molecular weight marker bands in kDa. Triangles and squares represent the dimeric and the monomeric form, respectively, of each protein.

Analysis by size exclusion chromatography (SEC) revealed that the Cry1Ia protoxin eluted in two peaks (Figure 3A). Peak 1 (P1) may correspond to a size of ~370 kDa (based on comparison to size standards) and possibly represent protoxin tetramers, while peak 2 (P2) of ~160 kDa corresponds to a dimeric form.

However, analysis by SDS-PAGE electrophoresis under non-denaturing conditions revealed that monomers (~80 kDa) and dimers (~160 kDa) were present in both P1 and P2 (inset in Figure 3A). Tetramers were not observed, probably because they were either not stable in the SDS-PAGE conditions used or because of their large molecular weight, which prevented resolving in SDS-12%PAGE gels. Interestingly, the dimeric state was relatively more abundant in P1, while the monomeric form was the major protein band in the P2 peak and subsequent fractions.

In the SEC chromatogram for the T-Int protein, three peaks (P1–3) and two shoulders (S1–S2) were observed (Figure 3B). P1 and S1 may correspond to aggregates (~1500 kDa and ~600 kDa, respectively), while the second peak (P2) could be attributed to tetramers or trimers (~240 kDa), and the size interpolated for proteins eluting in the S2 shoulder (~140 kDa) would correspond to the dimer state. P3 corresponds to proteins of ~15 kDa, probably representing peptides resulting from the processing of T-Int. Non-denaturing electrophoresis (inset in Figure 3B) showed doublet bands for both the monomeric and dimeric forms. This profile could be due to the different unfolding states of the proteins under the electrophoresis conditions used affecting the electrophoretic mobility of the different T-Int oligomeric forms.

The SEC chromatogram for the GF-Int protein exhibited an irregular baseline that was similar to the Cry1Ia protoxin chromatogram, with two major elution peaks (P1 and P2) (Figure 3C). Proteins eluting in the P1 and P2 fractions corresponded to sizes of ~240 kDa (tetramer or trimer) and ~120 kDa (dimer), respectively. In non-denaturing SDS-PAGE, the GF-Int fractions displayed a doublet of bands in both oligomeric and monomeric forms (inset in Figure 3C), as observed for T-Int. However, in concordance with the protoxin SEC, the most abundant band in both P1 and the fraction between P1 and P2 (P1-P2) corresponded to the oligomeric state, whereas in fractions within P2 the peptide was mainly in its monomeric state.

The fully trypsinized Cry1Ia protein was also analyzed by SEC chromatography. The elution profile showing several peaks was very similar to the one previously obtained by Khorramnejad et al. [14], and the major peak corresponded to the MW of the monomers (P3 in Figure 3D, MW ~55 kDa). Other detected peaks corresponded to aggregates (P1, with a MW of ~700 kDa) and dimers (minor peak P2, with a MW of ~140 kDa), and two more minor peaks (P4 and P5) corresponded to small peptides (~17 and ~5 kDa, respectively) (Figure 3D). While monomeric forms were expected only for the peak corresponding to the monomers (the major peak), non-denaturing SDS-PAGE analysis showed that monomers were detected in the first three peaks. This observation suggests that the interactions between the monomers resulting in any of the multimeric forms observed in the chromatogram were weak and probably due to aggregation.

### 2.3. Time Course of Cry1Ia Processing

**Figure 4:**
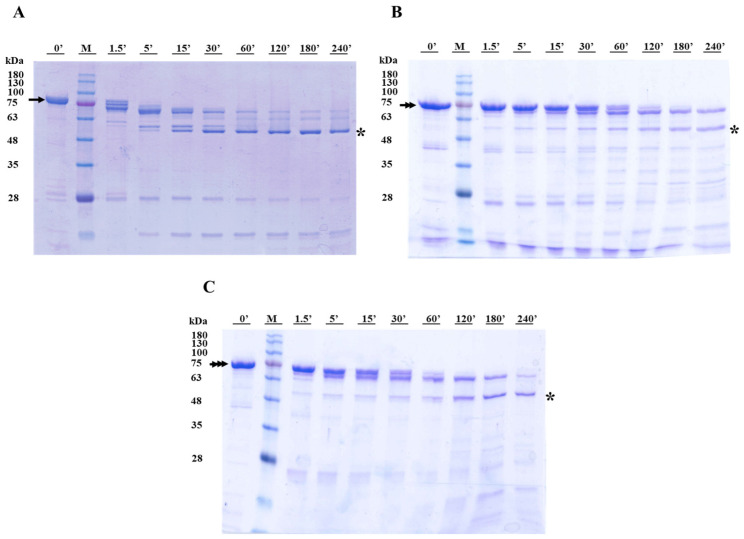
Processing of Cry1Ia protoxin (**A**), T-Int (**B**), and GF-Int (**C**) proteins with a 1:10 ratio of bovine trypsin to protein. Samples were resolved by SDS-PAGE and stained for total protein. Lanes 0′: Cry1Ia protein before trypsin addition; Lanes M: Blue Star marker (Nippon Genetics Europe GmbH, Düren, Germany); Lanes 1.5′ to 240′: time of processing with trypsin in minutes. The numbers on the left indicate the size of molecular weight marker bands in kDa. The protein bands of protoxin, T-Int, and GF-Int are indicated as simple, double, and triple arrows, respectively. The toxin bands are highlighted with an asterisk mark.

**Figure 5:**
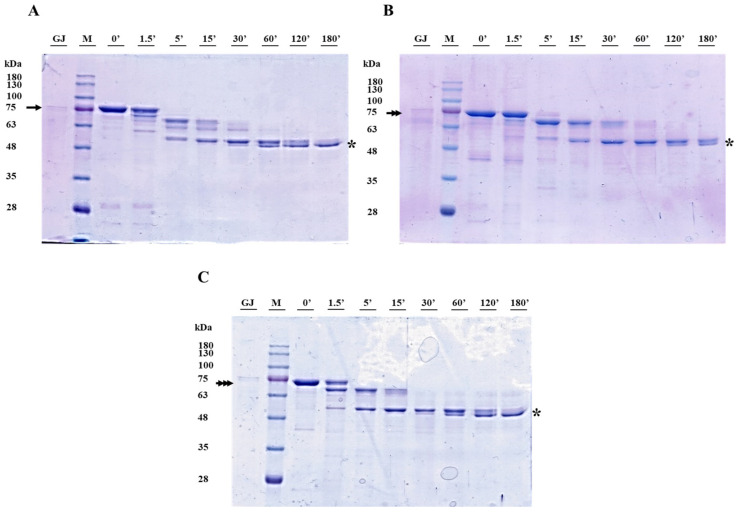
Processing of Cry1Ia protoxin (**A**), T-Int (**B**), and GF-Int (**C**) proteins with a 1:10 ratio of gut fluids from *O. nubilalis* larvae to protein. Samples were resolved by SDS-PAGE and stained for total protein. Proteins in gut fluids without Cry1Ia proteins are shown in lanes GJ. Lanes M: Blue Star marker (Nippon Genetics Europe GmbH, Düren, Germany); Lanes 0′: protein before larvae gut fluid addition; Lanes 1.5′ to 180′: time of protein incubation with larval gut fluids in minutes. The numbers on the left indicate the size of molecular weight marker bands in kDa. The protein bands of protoxin, T-Int, and GF-Int are indicated as simple, double, and triple arrows, respectively. The toxin bands are highlighted with an asterisk mark.

The processing of the Cry1Ia protoxin and intermediate forms was monitored using SDS-PAGE electrophoresis (Figure 4 and Figure 5). In the presence of trypsin, the Cry1Ia protoxin was sequentially processed over four hours to a ~50 kDa fully processed “toxin” form (Figure 4A). Three main protein bands were observed within 1.5 min of incubation. The smallest band of ~70 kDa, likely corresponding to the partially digested peptide T-Int, remained visible for up to 30 min of incubation. The fully processed toxin band (~50 kDa) appeared after 5 min, and it was the major band from 30 min onwards. Incubation of the T-Int and GF-Int proteins (Figure 4B,C) with trypsin also rendered the ~50 kDa toxin band, but through a slower process. Indeed, in both cases, a ~65 kDa band that appeared after about 30 min of processing coexisted with the fully activated toxin band even after 240 min of incubation. Despite the GF-Int and T-Int proteins differing by 11 amino acids at the C-terminus, these observations supported that proteolytic processing remained unaffected.

The processing of protoxin, T-Int, and GF-Int by gut fluids from *O. nubilalis* larvae was faster than that by processing with trypsin and produced the stable toxin core (~50 kDa) earlier (Figure 5). The proteolytic profiles for the three proteins were very similar throughout the assay, although the T-Int reaction only showed clear processing after more than 1.5 min. The ~50 kDa toxin band was observed after 5 min in all reactions and was the most abundant band after 30 min and the only band remaining after 180 min of processing.

### 2.4. Toxicity of Cry1I Protein Forms Against O. nubilalis and L. decemlineata

Surface contamination bioassays with *O. nubilalis* neonates showed the same toxicity for the Cry1Ia protoxin, T-Int, and GF-Int proteins, based on overlapping confidence intervals (Table 1). This suggested that the toxicity against *O. nubilalis* was not affected by the absence of amino acids in the C-terminus end, while the toxicity in the fully processed “toxin” was dramatically reduced.

**Figure 6:**
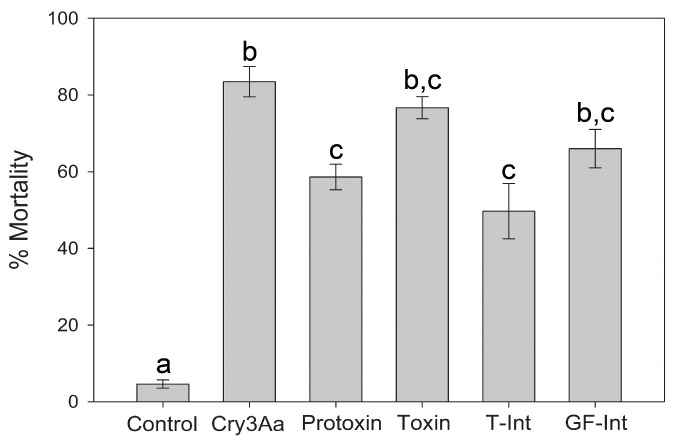
Toxicity of Cry1Ia protoxin (protoxin), fully processed toxin (toxin), and T-Int and GF-Int intermediate forms against *L. decemlineata* larvae. A discriminatory dose of 200 µg/mL was selected as producing 50–80% mortality in preliminary bioassays. Cry3Aa at 20 µg/mL was used as a positive control for mortality, and potato leaves coated with dilution buffer (tween-20) were used as a negative control for background mortality (<10%). The data shown are the means and corresponding standard errors from a minimum of two bioassays, each with 45 larvae. Different letters on top of columns represent significant differences (Kruskal–Wallis One Way ANOVA on Ranks for not normally distributed data with Dunn’s Method for multiple comparisons, *p* < 0.05).

In comparison, results from leaf-dipping bioassays with *L. decemlineata* larvae showed no significant differences (ANOVA on Ranks with Dunn’s Method for multiple comparisons, *p* < 0.05) between the toxicity of the Cry1Ia protoxin, T-Int, GF-Int, and fully trypsin-processed “toxin” forms (Figure 6). The detected significantly higher toxicity of a 10-fold lower concentration of Cry3Aa suggests that any of the Cry1Ia protein forms are relatively less active against *L. decemlineata* than the Cry3Aa protoxin.

## 3. Discussion

The intermediate products obtained during in vitro processing of Cry1I protoxins have been previously described [10,14,15,16], but their relative importance in the Cry1I mode of action has not been well established. Previous reports demonstrated that Cry1I protoxins have higher toxicity than fully activated toxin proteins in larvae of *O. nubilalis* [14,15] and *P. xylostella* [16], in contrast to similar or higher activity in toxin and protoxin observed with other Cry1 proteins against Lepidoptera [17,18,19,20]. In a previous study, the ~70 kDa protein obtained after partial trypsinization of the Cry1Ia protoxin retained most of the toxicity of the protoxin against *O. nubilalis* but was unstable and rapidly processed to the fully processed “toxin” form [14]. In the present study, we used Cry1Ia protoxin and T-Int and GF-Int truncated proteins representing processing intermediates in testing the role of proteolysis at the N- and C-terminal ends in the Cry1Ia intoxication process in lepidopteran and coleopteran species.

Typically found as monomers in solution [21,22,23,24,25,26], Cry proteins have also been observed in oligomeric states, such as tetramers [27]. Results from dynamic light scattering studies suggested that high pH and low salt conditions promoted Cry1Ac aggregation into dimers and trimers [28]. Previously, Cry1Ia protoxin was described to form dimers in solution [14], while the fully processed Cry1Ia protein was mostly present as monomers with dimers being less prevalent [14,16]. In the present study, analysis by SEC and non-denaturing SDS-PAGE revealed that the T-Int and GF-int intermediates are also capable of oligomerizing in solution into dimers and possibly tetramers, similarly to the protoxin and differently from the toxin, which remain mostly as monomers.

The first proteolytic cleavage at the C-terminus end of the Cry1Ia protoxin with trypsin occurs at R670 (T-Int) and at K659 with midgut fluids from *O. nubilalis* (GF-Int). In both cases, further proteolysis to the “toxin” form cleaves at R155 in the N-terminus end [14]. Processing of the Cry1Ia protoxin with trypsin was faster than the processing of the T-Int and GF-Int proteins, which resulted in a final ~65 kDa intermediate and the ~50 kDa fully processed “toxin” form, both resistant to further processing under the conditions tested. Further studies would be needed to sequence this ~65 kDa intermediate to determine additional protease cleavage sites in the protoxin that result in this form. Processing of all Cry1Ia protein forms tested by gut fluids from *O. nubilalis* was faster than with trypsin, probably due to the presence of multiple proteases in the *O. nubilalis* gut fluids that may assist with proteolysis. The ~65 kDa intermediate was noticeably less abundant when processing with gut fluids compared to trypsin, again suggestive of the participation of multiple gut proteases in the processing.

Both T-Int and GF-Int proteins were as toxic as the Cry1Ia protoxin against *O. nubilalis*, in contrast to the highly reduced activity observed for the ~50 kDa fully processed Cry1I “toxin”. This lack of activity in fully processed “toxin” has also been described for other Cry proteins. For example, chymotrypsin digestion of the ~130 kDa Cry9Ca1 protoxin resulted in a ~69 kDa intermediate protein toxic to *Spodoptera exigua*, which was further processed at Arg164 to yield the fully processed ~55 kDa inactive toxin form [29]. Similarly, the Cry2Aa1 protein (~60 kDa) is processed first to a ~58 kDa intermediate that retains toxicity against *Lymantria dispar*, but further processing at residue 144 in Domain I results in the fully processed (~50 kDa) and inactive toxin [30]. Complete processing of the Cry2Aa2 protoxin to toxin also eliminates activity against *Aedes aegypti*, which depends on the presence of residues within the first 49 amino acids at the N-terminus of the protoxin [31].

The observation that both T-Int and GF-Int proteins retained the solution state and toxicity against *O. nubilalis* that were observed for Cry1Ia protoxin supports that pre-processing at the C-terminus end does not affect interactions between monomers and toxicity. The activity of the Cry1Ia protoxin was not significantly different (based on overlapping confidence intervals) from our previous study [14]. However, in that study, a partially trypsinized Cry1Ia protoxin sample containing an intermediate form akin to the T-Int protein displayed slightly lower toxicity (~2.5-fold) to *O. nubilalis* compared to the Cry1Ia protoxin. The lack of differences in activity between Cry1Ia protoxin and T-Int in the present study, contrasting with the previous study, could be explained by the lower relative level of the T-Int intermediate in the partially trypsinized sample used before. As in *O. nubilalis*, the activity of the Cry1Ia, T-Int, and GF-Int proteins against larvae of *L. decemlineata* did not differ, supporting that the C-terminus is not needed for Cry1Ia toxicity in Lepidoptera and Coleoptera. This hypothesis is also supported by the stability of the Cry1Ie protein after the deletion of up to 86 amino acids from its C-terminus end [32]. In contrast, processing at the N-terminus end of Cry1Ia protoxin was shown to reduce or prevent oligomerization in solution [33] and impair toxicity against *P. xylostella* [16] and *O. nubilalis* ([14,15] and this study). In contrast, the Cry1Ia protoxin, intermediates, and fully trypsinized toxin were similarly active against *L. decemlineata* larvae.

Several studies have shown that the N-terminus end of Cry proteins plays a crucial role in the toxicity process, particularly in the formation of oligomeric structures and toxin pores in lepidopteran midgut cells [34]. Despite sharing sequence homology with other Cry1 proteins, Cry1I toxins exhibit significantly different proteolytic processing at their N-terminus end in lepidopteran hosts. In general, for Cry1A proteins, the cleavage site at the N-terminus is in amino acids 25 to 30 [35,36]. This cleavage was found to be essential for the insecticidal activity of the Cry1Ac protein against *Manduca sexta* larvae [37]. Processing of Cry2Ab protoxin to the toxin by midgut fluid proteases of *H. armigera* included cleavage at Arg139 between helices α-3 and α-4 in Domain I [35]. In contrast, the fully processed Cry1Ia protein is missing a fragment spanning helix α-1 to half of α-5 in the N-terminus, a region expected to be involved in oligomer formation [34]. This observation explains why the fully processed Cry1Ia toxin is less efficient at forming oligomers in solution and unable to form these structures in contact with BBMVs from *O. nubilalis* [14] and *Lobesia botrana* or with Sf21 cells [33], helping explain its lack of activity against lepidopteran larvae. Similarly, impaired oligomerization in Cry1Ab by a single point mutation in the N-terminus (R99E) resulted in a complete loss of toxicity against *M. sexta* larvae [38]. Mutations in residues located in the α4–α5 helices of domain I of the Cry2Ab protein were linked to less efficient pore-forming activity and reduced insecticidal activity against *P. xylostella* [39]. The α-3 and α-4 helices in the Cry9Aa protein are also critical in oligomerization and toxicity against *Chilo supressalis* [40].

The lack of differences in activity between Cry1Ia protoxin and toxin against *L. decemlineata* suggests that cleavage of the first 155 amino acids in the protoxin does not affect toxicity against coleopteran species. This observation supports that the Cry1Ia intoxication process may differ between hosts from distinct taxonomic orders, which is in line with current knowledge of the Cry mode of action in Coleoptera. For instance, Cry3Aa protoxin in *L. decemlineata* is cleaved at the N-terminus by an ADAM metalloprotease as part of the intoxication process [41], while in Lepidoptera, interactions with ADAM proteases have not been described. Moreover, cadherin proteins that are critical for the oligomerization of Cry toxins in Lepidoptera do not seem to be critical for Cry3Aa toxicity in *L. decemlineata* [42]. Future research should focus on further characterizing the mode of action of Cry1Ia proteins in hosts of distinct taxonomic orders to identify and engineer the optimal protein for the highest cross-order toxicity, guiding the development of pest control applications.

## 4. Conclusions

The results of this study show that the C-terminus in Cry1Ia protoxin is not necessary for toxicity against lepidopteran or coleopteran larvae. In contrast, a fragment that spans helix α-1 to half of α-5 in the N-terminus of the Cry1Ia protein is essential for toxicity against *O. nubilalis* but not against *L. decemlineata*. This information guides the design of new *cry1I* genes with cross-order toxicity through the inhibition of complete processing. These new genes could be transformed into transgenic plants to enhance pest management strategies.

## 5. Materials and Methods

### 5.1. Cry1Ia Site-Directed Mutagenesis (SDM)

The *cry1Ia38* gene (GenBank acc. number MG584186) was used in this study, and for clarity, it is referred to henceforth as “Cry1Ia”. The full-length *cry1Ia* gene was cloned into the pET-30a(+) vector containing a *kanamycin* (*kan*) resistance gene and an N-terminus histidine tag [43]. This clone was used as a template to obtain the Cry1I proteolytic intermediates by introducing stop codons using the SDM technique [44]. Several primer pairs (Table 2) were designed to insert stop codons in the Cry1Ia sequence in the position of residue V660 to create the intermediate form resulting from digestion with gut fluids (GF-Int) and in the position of the residue G671 for the trypsin proteolytic intermediate (T-Int) (Figure 1) [14]. Amplification was performed using KAPA HiFi DNA polymerase (KAPA Biosystems Pty, Cape Town, South Africa) in a thermal cycler (Eppendorf Mastercycler; Eppendorf, Hamburg, Germany) with the following conditions: initial denaturation for 3 min at 95 °C, 16 cycles of annealing for 30 s with variable temperature depending on the primers’ melting temperature, and a final extension for 15 min at 72 °C. Amplicons were confirmed using 1% agarose gel electrophoresis and then treated with *DpnI* enzyme (ThermoScientific Baltics, UAB, Vilnius, Lithuania) following the manufacturer’s recommendations, before ligation into pET-30a(+) following Khorramnejad et al. [43].

### 5.2. Expression and Purification of Cry1Ia Proteins

Competent DH10β *Escherichia coli* cells were transformed with 200 ng of each plasmid solution described in the previous section, containing either the *cry1Ia* gene, the *GF-Int* gene, or the *T-Int* gene. Successful transformants were selected on LB plates supplemented with 50 µg/mL kanamycin, and the plasmid DNA was purified from individual colonies using the NucleoSpin^®^ Plasmid kit (Macherey-Nage, Düren, Germany), according to the manufacturer’s instructions. Successful transformants were confirmed by sequencing (Stab Vida, Investigação e Serviços em Ciências Biologicas, Lisboa, Portugal). For expression, the *E. coli* BL21 (DE3) was used following procedures described elsewhere [43]. Briefly, transformants producing the Cry1Ia protoxin, T-Int, or GF-Int proteins were grown in LB supplemented with 50 µg/mL kanamycin for 16 h at 37 °C and 180 rpm. The precultures were inoculated into 750 mL of 2 × TY (16% tryptone, 10% yeast extract, 5% NaCl; *w*/*v*) supplemented with 50 µg/mL kanamycin at a 1:100 (preculture/culture) ratio and incubated at 37 °C and 180 rpm until the OD600 reached 0.5–0.6. Subsequently, expression was induced by the addition of 1 mM IPTG (isopropylthio-β-D-galactoside; Fisher bioreagents, Geel, Belgium) and incubation for 2 h at 25 °C and 180 rpm, after which 375 mL of Phosphate Buffered Saline (PBS; Fisher bioreagents, Geel, Belgium) were added to the cultures. The cultures were then centrifuged for 15 min at 4 °C, 15,000× *g*, and the pelleted cell mass was stored for 16 h at −80 °C. Lysis buffer (10 mL of lysis buffer per gram of pellet) composed of PBS (pH 7.5) plus 40 mM imidazole, 0.2 mg/mL lysozyme (PanReac AppliChem, Darmstadt, Germany), 20 µg/mL DNaseI (Roche Diagnostics GmbH, Mannheim, Germany), and 1 mM p-APMSF (p-amidinophenylmethylsulfonyl fluoride; Sigma-Aldrich, St. Louis, MO, USA) was used to resuspend pellets, and the mixture was incubated for 30 min at room temperature with gentle shaking. After sonication (Bandelin SONOPLUS HD 2200; BANDELIN electronic GmbH & Co. KG, Berlin, Germany) for 10 cycles (1 min of sonication with 1 min hold at 50% potency), the mixture was centrifuged for 15 min at 4 °C, 16,000× *g*. The supernatant was loaded onto a HisTrapTM FF Crude column (GE Healthcare Bio-Sciences, Uppsala, Sweden) connected to a MasterflexTM L/STM peristaltic pump (Fisher Scientific, Madrid, Spain) for protein purification according to the manufacturer’s instructions. The eluted purified sample was checked on SDS-12%PAGE electrophoresis [45]. The proteins were dialyzed in carbonate buffer (50 mM NaHCO_3_, 100 mM NaCl, pH 10.5) with Slide-A-Lyzer^®^ Dialysis Cassettes (ThermoScientific, Rockford, IL, USA), aliquoted, and stored at −20 °C until used.

To obtain the fully processed Cry1Ia toxin (~50 kDa), the purified Cry1Ia protoxin (~80 kDa) was quantified by the Bradford method [46] and mixed with TPCK-treated trypsin from bovine pancreas (Sigma-Aldrich, St. Louis, MO, USA) at a 1:10 (trypsin/protoxin) mass ratio. The sample was incubated for 4 h at 37 °C, and the complete processing was confirmed by SDS-12%PAGE.

The Cry3Aa protoxin was produced in cultures of *B. thuringiensis* subsp. *morrisoni* Biovar *tenebrionis* (*Bacillus* Genetic Stock Center, Columbus, OH, USA) and purified by anion exchange chromatography as described elsewhere [47]. Purified Cry3Aa protoxin was quantified using fluorometry (Qubit, Invitrogen) and preserved at −80 °C until used.

### 5.3. Size Exclusion Chromatography of Cry1Ia Forms

To elucidate the oligomeric state of the protoxin, T-Int, and GF-Int forms in solution, we performed an SEC analysis, as previously described [14]. The purified proteins were loaded on a Superdex-200 10/300 GL column (GE Healthcare Life Sciences, Uppsala, Sweden) pre-equilibrated with protein standards (44 kDa 4 mg/mL ovalbumin, 75 kDa 3 mg/mL conalbumin, 158 kDa 4 mg/mL ovalbumin, 440 kDa 0.3 mg/mL ferritin, and 669 kDa 5 mg/mL thyroglobulin) to estimate the molecular weight of the fractions eluted using an ÄKTA Explorer 100 chromatography system (GE Healthcare Life Sciences, Uppsala, Sweden). The proteins in the eluted peaks were examined by SDS-12%PAGE with loading buffer without SDS or β-mercaptoethanol and heated at 50 °C for 3 min to preserve oligomers. Purified protein samples before SEC purification were used as controls.

### 5.4. Time Course Proteolysis

The purified Cry1Ia proteins (protoxin, T-Int, and GF-Int) were processed in vitro according to the previously described methodology [14,48]. Purified Cry1I proteins were processed by trypsin (TPCK-treated bovine pancreas; Sigma-Aldrich, St. Louis, MO, USA) at a 1:10 (trypsin/protein) mass ratio and incubated at 37 °C for up to 240 min. Processing with gut fluids was performed with gut fluids obtained from dissected guts of last instar *O. nubilalis* larvae and then incubated with Cry1I proteins in a 1:10 mass ratio of gut fluids to protein at 30 °C for up to 180 min. After incubation, all processing samples were heat-denatured at 99 °C for 10 min and flash-frozen in liquid nitrogen until used for SDS-12%PAGE. Gels were stained for total protein with 0.1% (*v*/*v*) glacial acetic acid (C_2_H_4_O_2_; J.T. BakerTM, Gliwice, Poland), 0.45% (*v*/*v*) methanol (CH_3_OH; Labkem, Barcelona, Spain), and 1 g/L Coomassie brilliant blue R-250 (Bio-Rad, Watford, UK). At least two biological replicates were conducted for each processing assay.

### 5.5. Toxicity Bioassays Against O. nubilalis and L. decemlineata

The *O. nubilalis* FR population used for bioassays was originally obtained from the Institut National de la Recherche Agronomique (INRA, Paris, France) and had been reared in the Department of Genetics at the University of Valencia (Spain) for more than 5 years without exposure to Cry proteins. The insecticidal activity of Cry1Ia protoxin, T-Int, and GF-Int was tested against *O. nubilalis* neonates using a surface contamination method. Briefly, 50 µL of serial dilutions of each one of the purified proteins in carbonate buffer were poured into individual wells of 128-well bioassay trays (Frontier Agricultural Science, Newark, DE, USA; 2 cm^2^ diameter of each well) filled with artificial diet [49]. As a negative control, wells with artificial diet were treated with 50 µL of the carbonate buffer used for toxin purification and dilution. After air-drying, a single larva was placed in each well, and after sealing with an adhesive cover, trays were incubated at 25 ± 1 °C, 60 ± 5% RH, and 16:8 (light/dark) photocycle for seven days. Mortality was analyzed using probit analysis [50] with the POLO-PC program (LeOra Software, Berkeley, CA, USA). For each protein, three independent bioassay replicates were conducted with 16 individuals tested per concentration.

The METT population of *L. decemlineata* has been previously described [47]. Eggs were collected from mating cages containing METT adults feeding on potato (Solanum tuberosum var. Desiree) plants in pots under 27–30 °C, 70% relative humidity, and 18 h/6 h (light/dark) photoperiod. Collected eggs were kept in plastic cups with perforated lids until hatching, when neonates were moved to new plastic cups and fed daily with untreated potato leaves and maintained in an incubator (Percival, Perry, IA) under the same environmental conditions as adults. Bioassays were performed exposing second instar *L. decemlineata* larvae to potato leaves dipped in 200 μg/mL toxin solutions in 0.1% Tween-20 as a wetting agent. Exposure to potato leaves coated with Cry3Aa protoxin (20 μg/mL) or 0.1% Tween-20 was used as positive and negative controls for mortality, respectively. Potato leaf disks (12 mm diameter) were submerged in each test solution for approximately 10 s. Leaves were air-dried for approximately 15 min before introducing them into a plastic cup to which a second instar *L. decemlineata* larva was added. Bioassay cups were incubated under the same conditions as the rearing of adults for a total of five days, when mortality was assessed. Leaves were completely consumed and replaced on day 3 with freshly coated ones. A minimum of 15 larvae were tested per treatment and bioassay, and bioassays were repeated at least six times.

## Figures and Tables

**Table 1:** Toxicity parameters for Cry1Ia protoxin, T-Int, GF-Int, and trypsin-activated Cry1Ia toxin towards *O. nubilalis* larvae. C.I. = confidence intervals, S.E. = standard error, and ND = no determined.

		95% C.I.			95% C.I.		
Protein	LC_50_ (ng/cm^2^)	Lower	Upper	LC_90_ (ng/cm^2^)	Lower	Upper	Slope ± S.E.
Protoxin	73	58	90	161	126	239	3.9 ± 0.7
T-Int	67	50	85	170	130	273	3.5 ± 0.7
GF-Int	71	53	91	193	145	312	3.0 ± 0.5
“Toxin” ^1^	2197	1508	4544	ND	ND	ND	2 ± 0.4

^1^ Performed in our laboratory (University of Valencia) with the same methodology and insect colony as reported in Khorramnejad et al. [14].

**Table 2:** Primer pairs used for TP-Ia38 and LJ-Ia38 site-directed mutagenesis (SDM) and sequencing. Changed nucleotides are marked with underlined letters. T: trypsin intermediate peptide (T-Int), GF: gut fluid peptide (GF-Int), UAA, UAG, and UGA: STOP codons, F: forward primer, R: reverse primer, Tm: melting temperature.

NAME	Sequence (5′-3′)	T_m_ (°C)
GF-UAA-F	CAGAATATGATTTTGAAAAAGCGCAAGAGAAGTAAACTGCACTGTTTACATCTACG	80.2
GF-UAA-R	CTTGGATTCGTAGATGTAAACAGTGCAGTTTACTTCTCTTGCGCTTTTTCAAAATC	81.5
GF-UAG-F	CAGAATATGATTTTGAAAAAGCGCAAGAGAAGTAGACTGCACTGTTTACATCTACG	80.5
GF-UAG-R	CTTGGATTCGTAGATGTAAACAGTGCAGTCTACTTCTCTTGCGCTTTTTCAAAATC	81.8
GF-UGA-F	CAGAATATGATTTTGAAAAAGCGCAAGAGAAGTGAACTGCACTGTTTACATCTACG	81.8
GF-UGA-R	CTTGGATTCGTAGATGTAAACAGTGCAGTTCACTTCTCTTGCGCTTTTTCAAAATC	83.0
T-UAA-F	GGTTACTGCACTGTTTACATCTACGAATCCAAGATAATTAAAAACAGATGTAAAGG	77.4
T-UAA-R	GGTCAATATGATAATCCTTTACATCTGTTTTTAATTATCTTGGATTCGTAGATGTAAACAG	76.7
T-UAG-F	GGTTACTGCACTGTTTACATCTACGAATCCAAGATAGTTAAAAACAGATGTAAAGG	77.6
T-UAG-R	GGTCAATATGATAATCCTTTACATCTGTTTTTAACTATCTTGGATTCGTAGATGTAAACAG	76.9
T-UGA-F	GGTTACTGCACTGTTTACATCTACGAATCCAAGATGATTAAAAACAGATGTAAAGG	78.9
T-UGA-R	GGTCAATATGATAATCCTTTACATCTGTTTTTAATCATCTTGGATTCGTAGATGTAAACAG	78.1
SEQ-Ia38-F	TCTTCAGGTAACGAAGTTTATATAG	56.6
SEQ-Ia38-R	CGTATTTAACTATCTCGAATAATTC	56.1

## Data Availability

The original contributions presented in this study are included in the article. Further inquiries can be directed to the corresponding authors.
